# Ex vivo fluorescence confocal microscopy combined with rapid immunofluorescence for intraoperative intestinal mapping in near-total intestinal aganglionosis: a pediatric proof-of-concept case report

**DOI:** 10.3389/fmedt.2026.1869954

**Published:** 2026-07-29

**Authors:** Donatella Di Fabrizio, Alessandra La Contana, Edoardo Bindi, Alba Cruccetti, Gaia Goteri, Giovanni Cobellis

**Affiliations:** 1 Pediatric Surgery Unit, Salesi Children’s Hospital, Polytechnic University of Marche, Ancona, Italy; 2 Department of Clinical and Molecular Sciences (DISCLIMO), Polytechnic University of Marche, Ancona, Italy; 3 Department of Specialized Clinical and Odontostomatological Sciences, Polytechnic University of Marche, Ancona, Italy; 4 Department of Biomedical Sciences and Public Health, Institute of Pathological Anatomy, Polytechnic University of Marche, Ancona, Italy

**Keywords:** ex-vivo fluorescence confocal microscopy, fresh-tissue imaging, Hirschsprung disease, near-total intestinal aganglionosis, rapid immunofluorescence

## Abstract

**Introduction:**

In Hirschsprung disease (HD), intraoperative identification of ganglionated bowel remains central to surgical planning, particularly in extensive or near-total intestinal aganglionosis (nTIA), where bowel preservation is critical. Frozen-section histopathology remains the intraoperative standard. Ex-vivo fluorescence confocal microscopy (FCM) provides microscopic assessment of fresh tissue, while rapid immunofluorescence (rIF) has the potential to add targeted visualization of enteric neural structures. We describe a single pediatric proof-of-concept case in which FCM combined with selected rIF was integrated into intraoperative intestinal mapping.

**Case-report:**

A full-term newborn, diagnosed at birth with nTIA, underwent tube stoma formation at 40 cm from the Treitz ligament without bowel resection. At 4-years, surgery was scheduled to remove aganglionic segments and lengthen the remaining bowel. During surgery the small bowel segment proximal to the ostomy measured 70 cm. Twenty-four full-thickness biopsies were collected every 20 cm from sigmoid colon to presumed ganglionic areas at stoma level. All samples underwent FCM for morphological assessment of fresh tissue. rIF was applied to four selected samples, two from presumed ganglionated bowel and two from presumed aganglionic bowel, as an adjunct to enhance visualization of S100B-positive neural and glial structures. Fresh tissue was incubated for 30 min with a primary anti-S100B antibody followed by an Alexa Fluor 647-conjugated secondary antibody, then rinsed and counterstained with acridine orange. Under fluorescence laser visualization ganglion cells were identified in functional segments while hypertrophic nerve fibers were detected in aganglionic regions. Surgical planning relied on integrated intraoperative assessment, with final confirmation by conventional histopathology. The simultaneous application of a longitudinal intestinal lengthening (LILT) ensured maximization of functional bowel.

**Conclusion:**

This single-case experience demonstrates the technical feasibility of integrating *ex vivo* FCM and rIF into intraoperative intestinal mapping for complex Hirschsprung disease. The approach should be regarded as exploratory and complementary to established histopathological assessment.

## Introduction

Hirschsprung disease (HD) is a congenital disorder of the enteric nervous system characterized by absence of ganglion cells in the distal bowel, resulting in functional intestinal obstruction (1, 2). Surgical treatment depends on accurate identification of the transition zone, as the extent of resection directly influences postoperative function and the risk of persistent symptoms (3). This is particularly critical in extensive or near-total intestinal aganglionosis, where preservation of functional bowel is essential to limit intestinal failure and short bowel syndrome (4, 5).

Intraoperative decision-making is currently based on serial biopsies with frozen-section histopathology (5, 6). In extensive intestinal aganglionosis, the need for multiple samples, prior surgery, altered anatomy, and limited residual bowel length increases the need for precise coordination between the surgical and pathology teams. In this setting, *ex vivo* fluorescence confocal microscopy (FCM), enabling high-resolution microscopic assessment of fresh tissue within minutes (7, 8), and enhancing visualization of tissue-specific structures when combined with rapid immunofluorescence (rIF) (9–11), could be a potential fresh-tissue imaging method for targeted visualization of neural-related structures when rapid and reliable real-time bowel mapping is particularly valuable.

We report the first intraoperative use of *ex vivo* FCM combined with rIF for intestinal mapping in a child with near-total intestinal aganglionosis and short bowel syndrome. In this setting, the technique was explored as an adjunctive fresh-tissue imaging approach to assist intraoperative intestinal mapping during definitive reconstructive surgery.

## Case report

A female infant born at term at 39 + 3 weeks’ gestation, with a birth weight of 2,620 g, presented on the first day of life with bilious vomiting and abdominal distension suggestive of intestinal obstruction. Initial imaging demonstrated gastric and proximal bowel dilatation, microcolon, and absence of rectal air. Given the maternal history of intestinal neuronal abnormality, a rectal suction biopsy was performed during the neonatal period and confirmed rectal aganglionosis.

Subsequent serial intraoperative biopsies revealed extensive aganglionosis involving the entire colon and distal small bowel, with ganglion cells identified only in the proximal jejunum. A tube jejunostomy was therefore created 40 cm distal to the Treitz ligament, while the aganglionic distal bowel was left *in situ*. Over time, the patient developed severe short bowel syndrome and intestinal failure, requiring prolonged parenteral nutrition via a central venous catheter and ongoing jejunostomy management. Genetic analysis identified a heterozygous EDNRB variant, also detected in the mother, consistent with autosomal dominant susceptibility to Hirschsprung disease. Her clinical course was further complicated by recurrent catheter-related sepsis and high stoma output, leading to repeated hospital admissions, fluid replacement, and long-term nutritional support.

At 4 years of age, definitive surgical revision was planned. Preoperative contrast studies showed marked dilatation of the residual proximal small bowel, measuring up to 5.5 cm in diameter and approximately 70 cm in length. Intraoperatively, the proximal bowel segment measured about 70 cm from the Treitz ligament, whereas the excluded distal bowel measured approximately 135 cm, including the entire colon. Because precise identification of the transition between ganglionic and aganglionic bowel was essential to preserve every possible centimeter of functional intestine, intraoperative intestinal mapping was performed using *ex vivo* fluorescence confocal microscopy (FCM).

Twenty-four full-thickness samples were collected according to a predefined intraoperative mapping strategy, every 20 cm from sigmoid colon to the proximal small bowel. All fresh full-thickness intestinal samples were processed immediately after surgical sampling and transferred to the intraoperative imaging station without fixation, freezing, paraffin embedding, or sectioning. *Ex vivo* fluorescence confocal microscopy was performed using the VivaScope 2500 M-G4 system, developed by MAVIG GmbH, Munich, Germany, and Caliber I.D., Rochester, NY, USA. The system is equipped with dual laser excitation at 488 nm for fluorescence imaging and 785 nm for reflectance imaging, with a maximum imaging depth of 200 μm, an axial resolution of approximately 4 μm, and a maximum scan area of 25 × 25 mm^2^. Images were acquired as 1,024 × 1,024-pixel tiles and automatically assembled into digital mosaics with pseudo-H&E visualization. For FCM-only assessment, tissue samples were gently rinsed in 0.9% saline solution to remove blood and debris, briefly immersed in 70% ethanol for 10 s, stained with 0.04% acridine orange for 30 s, counterstained with 0.067% fast green for 20 s, and rinsed again in 0.9% saline solution for 10 s. Each sample was then positioned on the dedicated support, covered with the magnetic-mounted glass slide, placed in contact with the objective using ultrasound gel, and scanned after manual selection of the area and depth of interest.

In order to test whether S100B-based fluorescence could enhance recognition of enteric neural-related structures, rIF was applied to four selected specimens, two from presumed ganglionated areas (proximal ostomy site) and two from presumed aganglionic areas (distal ostomy site).

These biopsies were primarily incubated in phosphate-buffered saline containing 1% Tween-20 with a primary anti-S100B antibody (1:100; Molecular Probes, Thermo Fisher Scientific, Waltham, MA, USA; Cat# MA5-12969) and an Alexa Fluor 647-conjugated secondary antibody (1:1000; Molecular Probes, Thermo Fisher Scientific, Waltham, MA, USA; Cat# A-21235) in a humid chamber at room temperature for approximately 30 min. After washing with 0.9% saline, samples were counterstained with 0.04% acridine orange for 30 s, rinsed again, and examined using the fluorescence laser mode of the FCM system.

Image interpretation was performed intraoperatively by an expert senior pathologist with expertise in FCM image evaluation. The interpretation integrated FCM morphology, S100B fluorescence distribution when rIF was performed, anatomical sampling site, and subsequent conventional histopathology. Frozen-section histopathology was not performed during this procedure and *ex vivo* FCM and rIF were used as intraoperatively imaging methods, therefore, this case does not establish superiority, equivalence, or replacement value compared with frozen section. Final histological examination after formalin fixation, paraffin embedding, sectioning, and H&E staining served as the reference standard.

**Figure 1:**
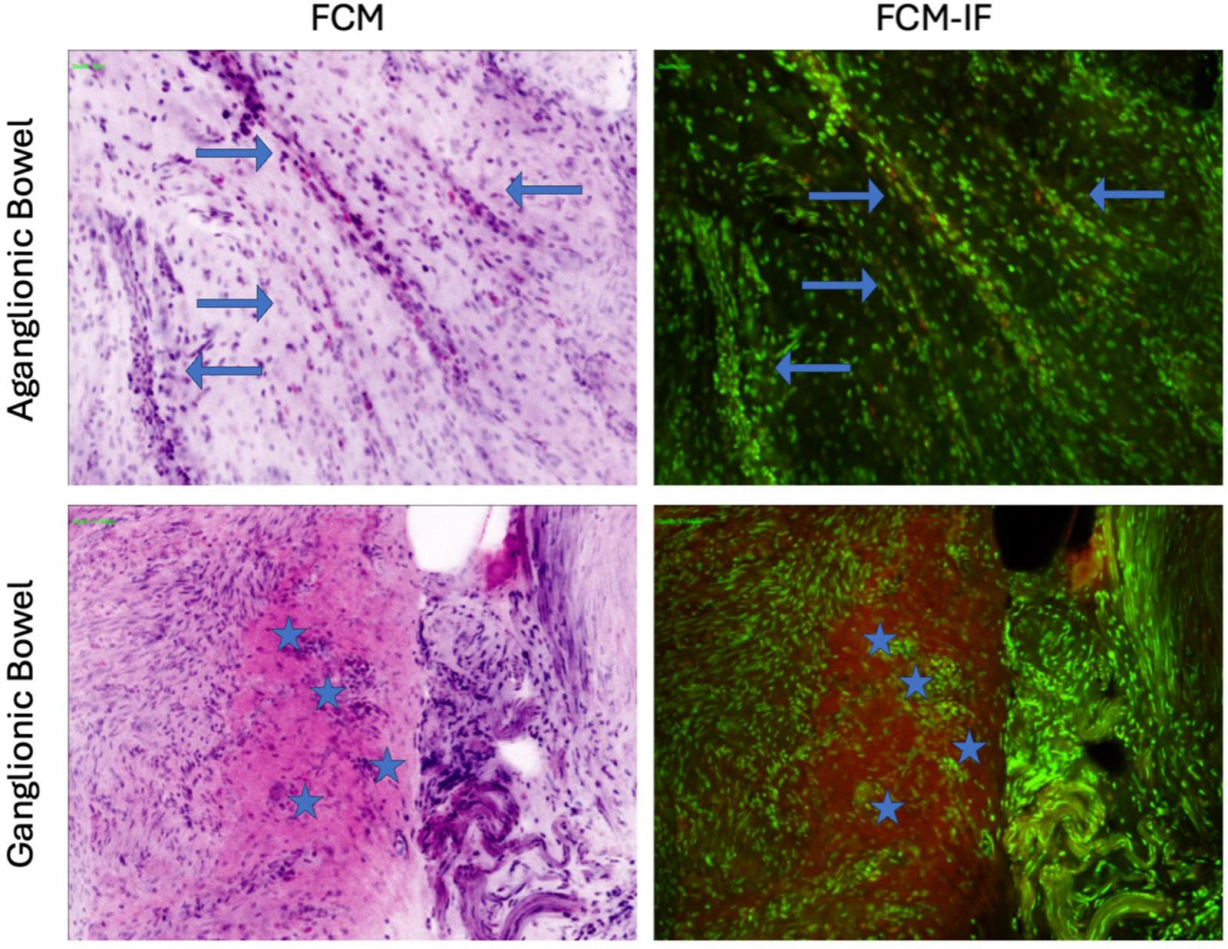
Representative *ex vivo* fluorescence confocal microscopy (FCM, left column) and fluorescence confocal microscopy with immunofluorescence (FCM-IF, right column) images of aganglionic bowel (top row) and ganglionic bowel (bottom row). The figure highlights the morphological and immunofluorescence differences between the two tissue types. In the FCM-IF images, the red signal corresponds to anti-S100B Alexa Fluor 647-conjugated antibody. In aganglionic bowel, FCM-rIF highlighted S100B-positive hypertrophic nerve fibers without identifiable ganglion cells (arrows). In ganglionated bowel, S100B staining outlined ganglion-associated glial structures in association with preserved enteric architecture (stars).

This combined approach enabled direct visualization of ganglion cells in functional bowel segments and hypertrophic nerve fibers in aganglionic tracts, thereby allowing sequential intraoperative mapping of the intestine (Figure 1). The confocal findings guided the definition of resection margins, leading to resection of the aganglionic distal bowel and of the distal non-functional portion of the proximal segment immediately upstream of the stoma, while preserving the maximum possible length of useful bowel. The patient subsequently underwent longitudinal intestinal lengthening and tailoring according to the Bianchi procedure (LILT), with refashioning of the proximal stoma. The postoperative course was uneventful. Histopathological examination confirmed complete concordance with the intraoperative confocal and immunofluorescence findings in this single case. At early postoperative follow-up, the child showed recovery of oral intake, weight gain, and continued management with cycling home parenteral nutrition.

## Discussion

This report describes, to our knowledge, the first intraoperative use of *ex vivo* FCM combined with rIF for intestinal mapping in a child with near-total intestinal aganglionosis and short bowel syndrome. The main finding is technical feasibility: fresh-tissue FCM was applied to multiple full-thickness intestinal samples during surgery, while S100B-based rIF was applied to four selected specimens to explore whether neural-related fluorescence labeling could improve visualization of ganglionic and aganglionic patterns. The observed findings were concordant with final histopathology in this individual case. Because this is a single case without blinded interpretation, standardized diagnostic thresholds, or quantitative performance metrics, the study should be interpreted as proof-of-concept rather than evidence of diagnostic accuracy.

In Hirschsprung disease, accurate definition of the transition zone is central to surgical success, particularly in extensive forms where diagnostic certainty must be balanced against preservation of limited absorptive bowel (1, 5, 6). Frozen-section histopathology remains the standard intraoperative method, but serial biopsies may be operationally demanding in complex reconstructions and previously operated abdomens (6, 12). In this context, *ex vivo* FCM offers rapid microscopic assessment of fresh tissue and can be integrated into the operative workflow as an adjunctive imaging approach (7, 8).

The innovation in this case lies in coupling FCM with rIF, a strategy reported mainly in dermatology (9–11) but with compelling biological and clinical potential in pediatric intestinal surgery. In our case, rIF enhanced visualization of enteric neural structures, enabling direct recognition of ganglion cells in functional bowel and hypertrophic nerve fibers in aganglionic segments. Immunofluorescent labeling for S100B, widely recognized as a marker of enteric glial cells and commonly employed in the study of Hirschsprung disease (13), combined with acridine orange nuclear counterstaining, provided high-resolution morpho-functional characterization of the tissue. S100B was selected because it labels enteric glial and peripheral glial elements associated with the enteric nervous system, providing a strong neural-related signal potentially suitable for rapid fluorescence imaging on fresh tissue. This choice differs from established immunohistochemical markers used in Hirschsprung disease, including neuronal markers such as HuC/D and PGP9.5, and diagnostic markers such as calretinin, which are commonly used to identify enteric neurons, nerve fibers, or ganglionated bowel. The rationale for S100B in the present workflow was technical and biological: it provided fluorescence signal compatible with fresh-tissue FCM-rIF and highlighted the glial framework around ganglionic structures as well as hypertrophic nerve fibers in aganglionic bowel.

This technically feasible approach helped the discrimination between ganglionated and aganglionic intestinal segments, facilitating the delineation of the boundary between functional and non-functional bowel, supporting intraoperative identification of the resection margin, highlighting the potential role of rIF as a diagnostic-support tool. This report has the intrinsic limitations of a single-case proof-of-concept study. rIF was applied only to four selected samples, while the remaining biopsies were assessed by FCM morphology alone. Intraoperative image interpretation was performed in a real surgical setting and was not designed as a blinded diagnostic accuracy study. Therefore, sensitivity, specificity, predictive values, and reproducibility cannot be inferred from this case. For these reasons, the findings should be interpreted as preliminary and require prospective validation with standardized criteria, systematic sampling, blinded readers, and direct comparison with frozen-section histopathology.

## Conclusion

*Ex vivo* FCM combined with selected S100B-based rIF was technically feasible for intraoperative intestinal mapping in this single case of near-total intestinal aganglionosis. The approach provided fresh-tissue morphological imaging and adjunctive visualization of neural-related structures, but it should be considered exploratory and complementary to established histopathological assessment. Prospective studies with systematic rIF sampling and direct comparison with frozen section are required before diagnostic performance or clinical utility is defined.

## Data Availability

The raw data supporting the conclusions of this article will be made available by the authors, without undue reservation.
